# The Impact of Telemedicine on Quality of Care for Patients with Diabetes After March 2020

**DOI:** 10.1007/s11606-021-07367-3

**Published:** 2022-01-28

**Authors:** Jacob K. Quinton, Michael K Ong, Catherine Sarkisian, Alejandra Casillas, Sitaram Vangala, Preeti Kakani, Maria Han

**Affiliations:** 1 Division of General Internal Medicine and Health Services Research, Department of Medicine, University of California, 1100 Glendon Ave, Suite 900, Los Angeles, CA 90024 USA; 2 David Geffen School of Medicine, University of California, Los Angeles, Los Angeles, CA USA; 3 Geriatrics Research Education and Clinical Center, Veterans Affairs Greater Los Angeles Healthcare System, Los Angeles, CA USA; 4 Department of Health Policy & Management, Fielding School of Public Health, University of California, Los Angeles, Los Angeles, CA USA; 5 VA Center for the Study of Healthcare Innovation, Implementation and Policy (CSHIIP), Los Angeles, CA USA

## Abstract

**Background:**

The impact of telemedicine on ambulatory care quality is a key question for policymakers as they navigate payment reform for remote care.

**Objective:**

To evaluate whether utilizing telemedicine in the first 9 months of the COVID-19 pandemic impacted performance on a diabetes quality of care measure for patients at a large academic medical center. We hypothesized care quality would reduce less among telemedicine users.

**Design:**

Quasi-experimental design using binomial logistic regression. Covariates included age, gender, race, ethnicity, type of insurance, hierarchical condition category score, primary language at the individual level, and zip code–level income.

**Participants:**

All adult patients younger than 75 years of age diagnosed with type 2 diabetes mellitus (*N* = 16,588) as of 3/19/2020 at a single academic health center.

**Interventions:**

Completion of one or more telemedicine encounters with an institutional primary care physician or endocrinologist between 3/19/2020 and 12/19/2020.

**Main Measures:**

The components met in a five-item composite measure of diabetes quality of care, as of patients’ last clinical encounter. Items were (1) systolic blood pressure less than 140 mmHg, (2) hemoglobin A1c less than 8.0%, (3) using a statin and (4) aspirin, and (5) tobacco non-use.

**Key Results:**

From the pre- to post-period, the probability of meeting any given component of the composite measure for patients only utilizing in-person care was 21% lower (OR, 95% CI 0.79; 0.76, 0.81) and for the telemedicine users 2% lower (OR 0.98; 0.85, 1.13). There was an increased likelihood of meeting any given component among telemedicine users compared to in-person care alone (OR 1.25; 1.08, 1.44).

**Conclusions:**

Patients with diabetes utilizing telemedicine performed similarly on a composite measure of diabetes care quality compared to before the pandemic. Those not utilizing telemedicine had reductions. Telemedicine use maintained quality of care for patients with diabetes during the first 9 months of the COVID-19 pandemic.

**Supplementary Information:**

The online version contains supplementary material available at 10.1007/s11606-021-07367-3.

## BACKGROUND

The coronavirus disease 19 (COVID-19) pandemic and the subsequent public health emergency (PHE) triggered a more than twenty-fold increase in telemedicine utilization over a 2-week period in March 2020 in order to maintain access to services while mitigating the threat of possible COVID-19 transmission. As in-person visits sharply declined, the proportion of ambulatory care delivered via telemedicine (defined as both audio-only and audio-video encounters) increased to peak at nearly half of all ambulatory encounters in June 2020.^1^ Subsequently, telemedicine encounters fell with the resumption of in-person care, but telemedicine visit volume persisted at nearly a quarter of total ambulatory encounters for the first 9 months of the pandemic. This “blend” of in-person and remote care in the ambulatory setting transformed office-based medical practice. The durability of this blended model of ambulatory care will depend on not just the changes in access,^1–4^ possible exacerbations of disparities,^5–9^ but also whether or not remote care is of the same quality as care delivered in person.

The expansion of telemedicine during the PHE was supported by many temporary waivers of telemedicine regulations by the Centers for Medicare and Medicaid Services (CMS) to increase capacity, expand workforce, reduce administrative burden, and otherwise expand telemedicine services.^10^ Telehealth has been previously demonstrated to improve chronic disease management^11–14^ for small pilots of patients with diabetes. A widely cited meta-analysis included more than forty randomized trials^15–18^ mostly used telemonitoring.^19^ Broad populations have been studied including a randomized intervention considering quality of care domains for veterans,^20^ and among older, racially diverse populations.^13^ Despite this evidence until the COVID-19-related PHE, there has not been broad, population-wide telemedicine utilization, and previous studies were subject to the generalizability issues inherent in smaller demonstrations.

The Medicare Payment Advisory Commission (MedPAC) has recommended continuation of coverage for telemedicine for 1–2 years after the end of the PHE in order to gather data on access, quality, and cost of care,^21^ which was recently incorporated in the most recent CMS physician fee scale.^22^ Our study objective was to evaluate the impact of remote audio-video encounter utilization on the quality of care for patients with diabetes at a large academic medical center in the first 9 months of the PHE.^23^

## METHODS

### Data Sources

We analyzed electronic medical record (EMR) data for all patients who were identified by our institutional population registry as having type 2 diabetes mellitus (DM) as of March 19, 2020. We abstracted the following variables from our institution’s electronic medical record (EMR) at the individual level: decade of age, gender, race, ethnicity, category of insurance (Medicare, commercial, Medicaid, or institutional managed care plan), systolic blood pressure, hemoglobin A1c, aspirin and/or statin prescription, smoking status, ambulatory visits to primary care, endocrinology, and other department of medicine visits, as well as hierarchical condition category (HCC) score. We also included income information from the American Communities Survey 2018 5-year estimates at the five-digit zip code level.

### Design and Study Period

We used a quasi-experimental (difference-in-differences) design. We defined our post-period to include the 9 months following the declaration of “safer-at-home” orders in Los Angeles County on March 19, 2020.^24^ We defined two sequential pre-periods of the same duration in the 18 months prior to the declaration to identify if pre-pandemic quality of care trends were parallel.

### Study Population and Exclusion Criteria

Our study population (*N* = 21,460) consisted of all patients identified by institutional clinicians as having type 2 diabetes mellitus when added to the electronic medical record problem list. This is verified for accuracy by the institutional quality team. Two physicians, one registered nurse, and two non-clinicians independently verify a random sample of patients identified as having type 2 diabetes mellitus. The study population was restricted to align with quality reporting standards, restricting to include adult patients under age 75 who are not currently enrolled in hospice care (*N* = 16,588).

### Study Variables

#### Quality Outcome

We defined our outcome for quality of care for patients with diabetes using the same domains as indicators included in the CMS Diabetes Composite quality measure from the CMS Measures Inventory.^25^ For our primary outcome, we evaluated the probability of meeting any given component in the five-component composite measure. The components are the last recorded value of (1)systolic/diastolic blood pressure less than 140/90 mmHg, (2) hemoglobin A1c less than 8.0%, (3) active prescription for statins and/or(4) aspirin or other anti-platelet agents if not contraindicated, and (5) tobacco non-users or those that quit during the study period.^23^ We made pre-specified changes to the composite for the purposes of our analysis. Due to limitations in our EMR, we did not exclude patients who may have a contraindication for anti-platelets or consider other anti-platelet agents (i.e., clopidogrel) as meeting this measure criterion, and did not analyze diastolic blood pressure as the proportion of patients with diastolic-only hypertension is negligible. We also did not exclude patients who became pregnant during the study period and were not able to ascertain deaths in our cohort.

#### Exposure and Control Group

We identified all included patients with at least one telemedicine visit in the post-period to either a primary care clinician (*N* = 6,405) or an endocrinologist (*N* = 2,251) to define our total exposure group (*N* = 7,581). Telemedicine visits are defined at our institution using the (EPIC/Clarity) visit types as recorded in our EMR, and distinguish between telephone encounters, new video visits, and return video visits. Visit type codes are unique to the EMR instance but available on request.

#### Covariates

We included the following covariates in addition to our primary regressor: decade of age, gender, race, ethnicity, category of insurance (Medicare, commercial, Medicaid, or institutional managed care plan), hierarchical condition category (HCC) score, all at the individual level. We also included income information from the American Communities Survey 2018 5-year estimates at the five-digit zip code level.

Our study protocol was approved by the UCLA Institutional Review Board via expedited review.

### Statistical Analysis

Using a binomial regression model, we estimated the effect of telemedicine use (at least one encounter in primary care or endocrinology) by comparing the likelihood of meeting any individual component of the diabetes composite outcome in the 9 months before and after March 2020 between the exposure and control group. All models included as covariates the sociodemographic and clinical characteristics described above. We performed several sensitivity analyses including (1) meeting all five component indicators of the diabetes composite measure, and sequentially fewer (4/5, 3/5, 2/5, and 1/5) indicators, (2) meeting four component indicators omitting the systolic blood pressure variable, which we hypothesized would be difficult to assess via telemedicine, and (3) a subgroup analysis of patients receiving Medicare benefits (either through disability or over the age of 65 years) and meeting 4/5 indicators given hypothesized challenges of older and disabled patients in accommodating new technology (Appendix 1). As a fourth sensitivity analysis to determine if there was a disproportionate quality impact on complex patients, we performed a subgroup analysis of patients meeting 4/5 outcome indicators among those with an HCC score of two or above, double that of the typical Medicare beneficiary, approximately in the top decile of complexity for our population (Appendix 1).

A significance level of 0.05 was used throughout, and analyses were performed using Stata 16c (StataCorp, College Station, TX). Our binomial regression model was specified by using the STATA command “binreg”, and all sensitivity analyses were using logistic regression (“logistic”).

## RESULTS

**Figure 1:**
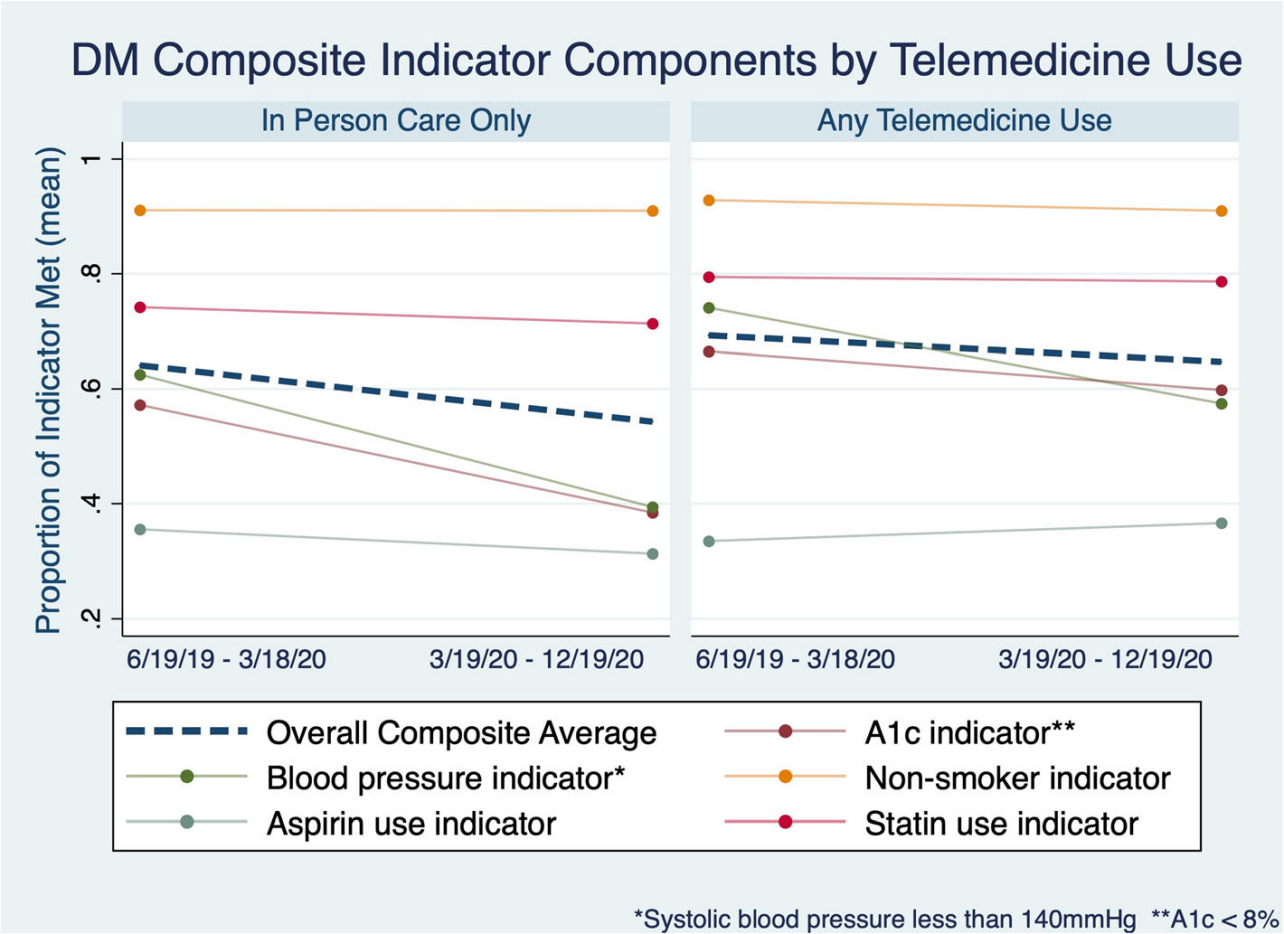
**DM composite indicator components by Telemedicine use.**

Very few patients with diabetes utilized telemedicine before the pandemic (*N* = 224, 1%) and most patients utilizing telemedicine in the post-period also had in-person appointments (6292, 86%). Both audio-only utilization (2,784, 17%) and audio-video utilization (7,357, 44%) increased dramatically in the post-period. Of audio-only utilizers, 66% were exclusively audio-only (i.e., no video encounters) telemedicine utilizers in the post-period. Telemedicine utilizers varied from those not utilizing telemedicine as less likely to have commercial insurance, more likely to be insured through Medicare and have lower HCC scores (Table 1). Nearly half of patients with diabetes utilized telemedicine during the first 9 months of the pandemic (*N* = 7,357, 44%). These patients were more likely to be female, Latino/a, more likely to be insured through Medicare and an institutional managed care plan, and have lower HCC scores (Table 1 and Fig. 1).

**Table 1:** Characteristics of patients with diabetes utilizing telemedicine compared to in-person care alone, in the 9 months before and after the beginning of the COVID-19 pandemic

**Characteristic**	Telemedicine utilizers		In-person care alone	
	Pre-period 6/19/19–3/19/20	Post-period 3/19/20–12/19/20	Pre-period 6/19/19–3/19/20	Post-period 3/19/20–12/19/20
Individuals (*N*)	224	7,357	16,364	9,231
Age, mean (SD)	60 (11)	60 (11)	60 (11)	60 (11)
Female (%)	119 (53)*	3,600 (49)*	7,348 (45)*	3,867 (42)*
Race (%)				
American Indian	1 ( < 1)	38 (1)	92 (1)	55 (1)
Asian	27 (12)	1,057 (14)	2,535 (15)	1,505 (16)
Black	21 (9)	822 (11)	1,605 (10)	804 (9)
Other/unknown	53 (24)	1,690 (23)	4,123 (25)	2,486 (27)
Pacific Islander	0 (0)	25 (< 1)	68 (< 1)	43 (< 1)
White	122 (54)	3,722 (51)	7,935 (49)	4.335 (47)
Ethnicity (% Latino/a)	51 (23)	1,402 (19)*	2,996 (18)	1,634 (18)*
Insurance type				
Commercial	86 (39)*	3,136 (43)*	7,584 (48)*	4,534 (52)*
Medicaid	1 (1)*	69 (1)*	180 (1)*	112 (1)*
Medicare	84 (38)*	2,530 (35)*	5,059 (32)*	2,613 (30)*
Other	1 (1)*	19 (< 1)*	38 (1)*	20 (1)*
Managed care	47 (21)*	1,514 (21)*	2,861 (18)*	1,394 (16)*
Primary language other than English (%)	10 (5)	497 (7)	1,185 (7)	698 (8)
HCC score				
0–1	163 (76)*	5,407 (74)*	11,379 (70)*	6,135 (66)*
1–2	29 (14) *	1,036 (14)*	1,681 (10)*	674 (7)*
2–3	10 (5) *	324 (4)*	510 (3)*	196 (2)*
3+	12 (6) *	267 (4)*	386 (2)*	131 (1)*
Missing	10 (4) *	323 (4)*	2,408 (15)*	2,095 (23)*
DM quality components				
A1c less than 8%	149 (67)*	4,401 (60)*	9,363 (58)*	3,549 (39)*
Systolic BP < 140	166 (76)*	4,228 (58)*	10,227 (63)*	3,639 (40)*
Statin prescription	178 (95)	5,788 (96)*	12,143 (95)	6,585 (95)*
Aspirin prescription	75 (84)	2,695 (82)	5,816 (87)	2,888 (83)
Tobacco non-use	208 (98)	6,695 (91)	14,908 (91)	8,401 (91)

*p < 0.05, comparisons between in-person care versus telemedicine users for each time period

Both groups had similar overall ambulatory utilization before and during the first 9 months of the pandemic (Appendix 2 Table 1). The proportion of patients meeting each component indicator of the overall diabetes composite measure were similar in the 18 months prior to the pandemic (Appendix 2, Fig. 1).

In adjusted estimates, patients utilizing in-person care alone in the first 9 months of the pandemic were less likely to meet any given component of the diabetes composite quality measure compared to before the pandemic (OR, 95% CI; 0.60, 0.56, 0.65), whereas patients utilizing telemedicine were no less likely (OR, 95% CI; 0.89, 0.65, 1.23). The interaction between time period and use of telemedicine services was significant (OR, 95% CI; 1.48, 1.07, 2.05), as well as the individual-level covariates age (by decade of life), gender, self-reported Asian or other race, and the zip code–level covariate income (by federal poverty level) (Table 2).

**Table 2:** Odds of meeting diabetes quality composite components among patients with diabetes utilizing telemedicine versus in-person care alone ^

**Characteristic**	**Odds ratio**	**95% confidence interval**	***p*value**
In-person care alone: post versus pre-period	0.60*	0.56–0.65	< 0.001
Any telemedicine use: post versus pre-period	0.89	0.65–1.23	0.484
Difference-in-differences	1.48*	1.07–2.05	0.02
Age (decade)	1.71*	1.66–1.78	< 0.001
Female gender	0.58*	0.55–0.62	< 0.001
Race or ethnic group			
Black	1.12	1.02–1.25	0.024
Asian	1.20*	1.10–1.30	< 0.001
Pacific Islander	1.23	0.79–1.94	0.36
American Indian	0.81	0.54–1.21	0.31
Other	0.90*	0.83–0.97	0.006
Insurance type			
Medicare	1.23	0.92–1.76	0.15
Commercial	1.15*	1.07–1.24	< 0.001
Managed care	1.94*	1.10–3.43	0.021
Medicaid	1.11*	1.02–1.20	0.008
Primary language	0.76	0.68–0.85	0.68
HCC score	1.18*	1.13–1.22	< 0.001

^Outcome is odds of meeting any additional quality indicator, binomial logistic regression, in the 9 months before and after the beginning of the COVID-19 pandemic

Patients utilizing telemedicine were as likely than those not using telemedicine to meet 5 of 5 components of the diabetes composite measure of quality of care when comparing the post-period to pre-period (OR, 95% CI; 1.27, 0.93, 1.73). Similarly, patients utilizing telemedicine were as likely as those using telemedicine to meet the remaining quality indicators when the blood pressure measurement component of the composite was excluded (1.21, 0.89, 1.66) (Appendix 1). Among the subgroup analysis of Medicare beneficiaries’ meeting 4 of 5 indicators (*N* = 9,264), we saw significant declines (OR 0.60; 0.54, 0.67) in both the in-person care and telemedicine utilizing groups (0.64; 0.41, 1.01) with non-significant differences between the groups (1.07; 0.68, 1.70). Similarly, among patients with an HCC score of 2 or more (*N* = 5,766), we observed a reduction in those meeting 4 of 5 indicators among patients receiving in-person care alone (0.45; 0.37, 0.55) although not among those patients utilizing telemedicine services (0.86; 0.38, 2.00), and the difference between the groups was not statistically significant (OR 1.91; 0.80, 4.57). Few patients with an HCC score of 2 or more utilized telemedicine (*N* = 946). Full covariate estimates for sensitivity analyses are available in a supplementary appendix (Appendix 1).

## DISCUSSION

Telemedicine has been shown to impact the quality of care for patients with diabetes in pre-pandemic demonstrations, but however has not previously been implemented across entire populations. Patients with diabetes in a large academic medical center who used telemedicine achieved similar quality outcomes compared to before the pandemic; however, patients who utilized only in-person care saw a decline in the quality outcome ascertainment. We found this to be the case using a composite indicator of quality of care for diabetes in the CMS measures inventory, despite care of similar quality during the 18 months before the pandemic, while controlling for an array of sociodemographic and clinical variables in our analysis. This demonstration of the incorporation of telemedicine as a mode of ambulatory care in addition to in-person visits during the early pandemic is an early indicator of the promise of telemedicine to deliver high-quality care across populations, during and beyond the public health emergency.

The largest differences between the telemedicine utilizing and the in-person care only group were seen in the indicators most likely to be impacted by a disruption of in-person care, i.e., blood pressure monitoring and laboratory testing (hemoglobin A1c), which may be improved with advances in remote patient monitoring. This disruption has been also seen in national cohorts. ^28^ The unanswered question is whether continued innovations in telemedicine as a mode of ambulatory care, including at the authors’ institution,^26^ will be able to improve the quality of care for populations over time. Recent single-payer evidence highlights the role of payment models in this innovation.^27^

Our study is not without limitations. First, our data were from a single institution in a large urban environment, and limitations to the use of telemedicine (such as broadband access or technology limitations) may be less prevalent in our study population than in a predominantly low-income or rural population. Second, our intervention included all patients with at least one telemedicine encounter in the 9 months following the pandemic, and while this was the first opportunity to study population-wide telemedicine implementation, the time was uniquely transitional as “safer at home” orders were enacted, vaccines were developing, and case rates in our home county and state varied widely, perhaps leading to unobserved variable biases not controlled for in our detailed model. Third, part of this first population-wide implementation of telemedicine is the acknowledgement that despite observed variable consistency in diabetes care among pre- and early-pandemic telemedicine utilizers, the population using telemedicine before the pandemic may have varied from the general population at our institution on unobservable characteristics.

In conclusion, the rapid expansion of telemedicine utilization has transformed ambulatory care delivery during the COVID-19 pandemic, and improved access to care to populations attempting to minimize exposure to a deadly respiratory pathogen. This unprecedented change in the mode of ambulatory care delivery has many unanswered questions currently pressing policymakers. We found that in a single institution, the utilization in the 9 months following the COVID-19 pandemic improved the quality of care for patients with diabetes. Future studies are needed to validate these results in state- or nation-wide populations, and with additional chronic diseases, in order to make evidence-based policy regarding the continuation of telemedicine coverage after the public health emergency concludes.

## Supplementary Information

ESM 1 (DOCX 39 kb)

ESM 2 (DOCX 7348 kb)
