# Supplementary material for: The Impact of Telemedicine on Quality of Care for Patients with Diabetes After March 2020
Source: J Gen Intern Med. 2022 Jan 28;37(5):1198–203. doi: 10.1007/s11606-021-07367-3 (PMC8796744; doi:10.1007/s11606-021-07367-3)
Supplement: Supplementary file 1 — (DOCX 39 kb) [file 11606_2021_7367_MOESM1_ESM.docx]

**Appendix 1**: Sensitivity Analyses

We defined our main analysis using the domains and indicators used in the diabetes composite measure. These were: 1) hemoglobin A1c less than 8%, 2) systolic blood pressure less than 140mmHg, 3) active prescription for aspirin and/or 4) statins if not contraindicated, and 5) tobacco non-users or those that quit during the study period. We included covariates consistent with our conceptual model and available in our institutional electronic medical record or available secondary datasets. These covariates included decade of age, gender, race, ethnicity, category of insurance, systolic blood pressure, hemoglobin A1c, aspirin and/or statin prescription, smoking status, ambulatory visits to primary care, endocrinology, and other department of medicine visits, hierarchical condition category (HCC) score, and zip-code level income, except when subgroup analyses obviated variable inclusion (for Medicare and HCC 2+ analyses).

**Sensitivity Analysis 1A**: Meets 5/5 criteria for diabetes quality composite measure (N=27,394)

| **Characteristic** | **Odds Ratio** | **95% Confidence Interval** | **p-value** |
| --- | --- | --- | --- |
| Control (In-person alone): Post versus Pre | 0.90* | 0.84 – 0.96 | 0.001 |
| Exposure (Telemedicine): Post versus Pre | 1.14 | 0.74 – 1.36 | 0.40 |
| Difference-in-Differences | 1.27 | 0.93 – 1.73 | 0.14 |
| Age (decade) | 1.73* | 1.68 – 1.79 | < 0.001 |
| Female Gender | 0.53* | 0.50 – 0.56 | < 0.001 |
| Race |  |  |  |
| Black compared to White | 1.16* | 1.06 – 1.27 | 0.002 |
| Asian compared to White | 1.04 | 0.96 – 1.12 | 0.36 |
| Pacific Islander compared to White | 1.07 | 0.70 – 1.62 | 0.76 |
| American Indian compared to White | 0.68* | 0.47 – 0.98 | 0.04 |
| Other compared to White | 0.90* | 0.84 – 0.96 | 0.003 |
| Ethnicity |  |  |  |
| Hispanic | 0.93 | 0.86 – 1.01 | 0.08 |
| Insurance Type |  |  |  |
| Medicaid compared to Commercial | 1.51* | 1.16 – 1.97 | 0.002 |
| Medicare compared to Commercial | 1.14* | 1.07 – 1.22 | < 0.001 |
| Other compared to Commercial | 2.08* | 1.21 – 3.59 | 0.008 |
| Managed care compared to Commercial | 0.99 | 0.92 – 1.06 | 0.72 |
| Primary language other than English | 0.69* | 0.62 – 0.76 | < 0.001 |
| HCC score | 1.21* | 1.17 – 1.26 | < 0.001 |
| Income, zip code level (FPL) | 1.00 | 0.98 – 1.02 | 0.89 |

**Sensitivity Analysis 1B**: Meets 4/5 criteria for diabetes quality composite measure (N=27,394)

| **Characteristic** | **Odds Ratio** | **95% Confidence Interval** | **p-value** |
| --- | --- | --- | --- |
| Control (In-person alone): Post versus Pre | 0.60* | 0.56 – 0.65 | < 0.001 |
| Exposure (Telemedicine): Post versus Pre | 0.89 | 0.65 – 1.23 | 0.484 |
| Difference-in-Differences | 1.48* | 1.07 – 2.05 | 0.02 |
| Age (decade) | 1.71* | 1.66 – 1.78 | < 0.001 |
| Female Gender | 0.58* | 0.55 – 0.62 | < 0.001 |
| Race |  |  |  |
| Black compared to White | 1.12 | 1.02 – 1.25 | 0.024 |
| Asian compared to White | 1.20* | 1.10 – 1.30 | < 0.001 |
| Pacific Islander compared to White | 1.23 | 0.79 – 1.94 | 0.36 |
| American Indian compared to White | 0.81 | 0.54 – 1.21 | 0.31 |
| Other compared to White | 0.90* | 0.83 – 0.97 | 0.006 |
| Ethnicity |  |  |  |
| Hispanic | 1.23 | 0.92 – 1.76 | 0.15 |
| Insurance Type | 1.15* | 1.07 – 1.24 | < 0.001 |
| Medicaid compared to Commercial | 1.94* | 1.10 – 3.43 | 0.021 |
| Medicare compared to Commercial | 1.11* | 1.02 – 1.20 | 0.008 |
| Other compared to Commercial | 0.76 | 0.68 - 0.85 | 0.68 |
| Managed care compared to Commercial | 1.18* | 1.13 - 1.22 | < 0.001 |
| Primary language other than English | 1.01* | 1.00 – 1.02 | 0.01 |
| HCC score | 0.60* | 0.56 – 0.65 | < 0.001 |
| Income, zip code level (FPL) | 0.89 | 0.65 – 1.23 | 0.484 |

**Sensitivity Analysis 1C**: Meets 4/5 criteria for diabetes quality composite measure (N=27,394)

| **Characteristic** | **Odds Ratio** | **95% Confidence Interval** | **p-value** |
| --- | --- | --- | --- |
| Control (In-person alone): Post versus Pre | 0.81 | 0.76 – 0.87 | < 0.001 |
| Exposure (Telemedicine): Post versus Pre | 1.05 | 0.78 – 1.32 | 0.733 |
| Difference-in-Differences | 1.30 | 0.95 – 1.77 | 0.104 |
| Age (decade) | 1.74 | 1.69 – 1.80 | < 0.001 |
| Female Gender | 0.54 | 0.51 – 0.57 | < 0.001 |
| Race |  |  |  |
| Black compared to White | 1.14 | 1.04 – 1.25 | 0.006 |
| Asian compared to White | 1.08 | 1.01 – 1.17 | 0.048 |
| Pacific Islander compared to White | 1.10 | 0.72 – 1.67 | 0.664 |
| American Indian compared to White | 0.76 | 0.53 – 1.09 | 0.141 |
| Other compared to White | 0.90 | 0.84 – 0.97 | 0.003 |
| Ethnicity |  |  |  |
| Hispanic | 0.96 | 0.89 – 1.04 | 0.363 |
| Insurance Type |  |  |  |
| Medicaid compared to Commercial | 1.45 | 1.11 – 1.89 | 0.007 |
| Medicare compared to Commercial | 1.15 | 1.07 – 1.23 | < 0.001 |
| Other compared to Commercial | 2.02 | 1.17 – 3.48 | 0.012 |
| Managed care compared to Commercial | 1.01 | 0.94 – 1.09 | 0.775 |
| Primary language other than English | 0.68 | 0.61 – 0.75 | < 0.001 |
| HCC score | 1.18 | 1.14 – 1.23 | < 0.001 |
| Income, zip code level (FPL) | 1.00 | 0.99 – 1.01 | 0.436 |

**Sensitivity Analysis 1D**: Meets 2/5 criteria for diabetes quality composite measure (N=27,394)

| **Characteristic** | **Odds Ratio** | **95% Confidence Interval** | **p-value** |
| --- | --- | --- | --- |
| Control (In-person alone): Post versus Pre | 0.89 | 0.83 – 0.95 | < 0.001 |
| Exposure (Telemedicine): Post versus Pre | 1.13 | 0.83 – 1.53 | 0.446 |
| Difference-in-Differences | 1.27 | 0.93 – 1.73 | 0.133 |
| Age (decade) | 1.73 | 1.69 – 1.79 | < 0.001 |
| Female Gender | 0.53 | 0.50 – 0.56 | < 0.001 |
| Race |  |  |  |
| Black compared to White | 1.16 | 1.06 – 1.27 | 0.002 |
| Asian compared to White | 1.04 | 0.97 – 1.13 | 0.262 |
| Pacific Islander compared to White | 1.08 | 0.71 – 1.64 | 0.720 |
| American Indian compared to White | 0.69 | 0.48 – 0.90 | 0.044 |
| Other compared to White | 0.90 | 0.84 – 0.96 | 0.002 |
| Ethnicity |  |  |  |
| Hispanic | 0.93 | 0.86 – 1.01 | 0.073 |
| Insurance Type |  |  |  |
| Medicaid compared to Commercial | 1.48 | 1.13 – 1.93 | 0.004 |
| Medicare compared to Commercial | 1.14 | 1.06 – 1.22 | < 0.001 |
| Other compared to Commercial | 2.10 | 1.22 – 3.62 | 0.007 |
| Managed care compared to Commercial | 0.99 | 0.92 – 1.06 | 0.730 |
| Primary language other than English | 0.68 | 0.61 – 0.76 | < 0.001 |
| HCC score | 1.21 | 1.16 – 1.26 | < 0.001 |
| Income, zip code level (FPL) | 1.00 | 0.99 – 1.01 | 0.920 |

**Sensitivity Analysis 1E**: Meets 1/5 criteria for diabetes quality composite measure (N=27,394)

| **Characteristic** | **Odds Ratio** | **95% Confidence Interval** | **p-value** |
| --- | --- | --- | --- |
| Control (In-person alone): Post versus Pre | 0.90 | 0.84 – 0.96 | 0.001 |
| Exposure (Telemedicine): Post versus Pre | 1.13 | 0.74 – 1.36 | 0.992 |
| Difference-in-Differences | 1.27 | 0.93 – 1.73 | 0.135 |
| Age (decade) | 1.73 | 1.68 – 1.79 | < 0.001 |
| Female Gender | 0.53 | 0.50 – 0.56 | < 0.001 |
| Race |  |  |  |
| Black compared to White | 1.16 | 1.06 – 1.27 | 0.002 |
| Asian compared to White | 1.04 | 0.96 – 1.12 | 0.361 |
| Pacific Islander compared to White | 1.07 | 0.70 – 1.61 | 0.760 |
| American Indian compared to White | 0.68 | 0.47 – 0.98 | 0.038 |
| Other compared to White | 0.90 | 0.84 – 0.96 | 0.003 |
| Ethnicity |  |  |  |
| Hispanic | 0.93 | 0.86 – 1.01 | 0.076 |
| Insurance Type |  |  |  |
| Medicaid compared to Commercial | 1.51 | 1.16 – 1.98 | 0.002 |
| Medicare compared to Commercial | 1.14 | 1.07 – 1.22 | < 0.001 |
| Other compared to Commercial | 2.08 | 1.21 – 3.59 | 0.008 |
| Managed care compared to Commercial | 0.99 | 0.92 – 1.06 | 0.723 |
| Primary language other than English | 0.69 | 0.62 – 0.76 | < 0.001 |
| HCC score | 1.21 | 1.17 – 1.26 | < 0.001 |
| Income, zip code level (FPL) | 1.00 | 0.99 – 1.01 | 0.891 |

**Sensitivity Analysis 2**: Meeting full composite criteria without systolic blood pressure indicator (N=27,394)

| **Characteristic** | **Odds Ratio** | **95% Confidence Interval** | **p-value** |
| --- | --- | --- | --- |
| Control (In-person alone): Post versus Pre | 0.89* | 0.84 – 0.95 | < 0.001 |
| Exposure (Telemedicine): Post versus Pre | 1.09 | 0.80 – 1.47 | 0.59 |
| Difference-in-Differences | 1.21 | 0.89 – 1.66 | 0.21 |
| Age (decade) | 1.74* | 1.69 – 1.80 | < 0.001 |
| Female Gender | 0.52* | 0.50 – 0.55 | < 0.001 |
| Race |  |  |  |
| Black compared to White | 1.17* | 1.06 – 1.28 | 0.001 |
| Asian compared to White | 1.02 | 0.95 – 1.10 | 0.571 |
| Pacific Islander compared to White | 1.14 | 0.76 – 1.73 | 0.527 |
| American Indian compared to White | 0.69* | 0.48 – 0.99 | 0.042 |
| Other compared to White | 0.89* | 0.84 – 0.96 | 0.002 |
| Ethnicity |  |  |  |
| Hispanic | 0.933* | 0.86 – 1.01 | 0.08 |
| Insurance Type |  |  |  |
| Medicaid compared to Commercial | 1.51* | 1.15 – 1.96 | 0.003 |
| Medicare compared to Commercial | 1.14* | 1.07 – 1.23 | < 0.001 |
| Other compared to Commercial | 2.04* | 1.19 – 3.51 | 0.01 |
| Managed care compared to Commercial | 0.99 | 0.92 – 1.06 | 0.72 |
| Primary language other than English | 0.68* | 0.61 – 0.75 | < 0.001 |
| HCC score | 1.22* | 1.17 – 1.27 | < 0.001 |
| Income, zip code level (FPL) | 1.00 | 0.98 – 1.02 | 0.91 |

**Sensitivity Analysis 3**: Meets 4/5 criteria for DM composite if Medicare beneficiary (N=9,264)

| **Characteristic** | **Odds Ratio** | **95% Confidence Interval** | **p-value** |
| --- | --- | --- | --- |
| Control (In-person alone): Post versus Pre | 0.60* | 0.54 – 0.67 | < 0.001 |
| Exposure (Telemedicine): Post versus Pre | 0.64 | 0.41 – 1.01 | 0.06 |
| Difference-in-Differences | 1.07 | 0.68 – 1.70 | 0.77 |
| Age (decade) | 1.35* | 1.27 – 1.44 | < 0.001 |
| Female Gender | 0.60* | 0.55 – 0.66 | < 0.001 |
| Race |  |  |  |
| Black compared to White | 1.22* | 1.05 – 1.42 | 0.01 |
| Asian compared to White | 1.28* | 1.12 – 1.46 | < 0.001 |
| Pacific Islander compared to White | 0.35* | 0.10 – 1.21 | 0.01 |
| American Indian compared to White | 0.73 | 0.37 – 1.43 | 0.36 |
| Other compared to White | 1.04 | 0.92 – 1.17 | 0.52 |
| Ethnicity |  |  |  |
| Hispanic | 1.05 | 0.91 – 1.20 | 0.51 |
| Primary language other than English | 0.75* | 0.65 – 0.88 | < 0.001 |
| HCC score | 1.07* | 1.02 – 1.13 | 0.01 |
| Income, zip code level (FPL) | 1.03* | 1.00 – 1.06 | 0.04 |

**Sensitivity Analysis 4**: Meets 4/5 criteria for DM composite if HCC score of 2+ (N=5,766)

| **Characteristic** | **Odds Ratio** | **95% Confidence Interval** | **p-value** |
| --- | --- | --- | --- |
| Control (In-person alone): Post versus Pre | 0.45* | 0.37 – 0.55 | < 0.001 |
| Exposure (Telemedicine): Post versus Pre | 0.86 | 0.38 – 2.00 | 0.72 |
| Difference-in-Differences | 1.91 | 0.80 – 4.57 | 0.14 |
| Age (decade) | 1.53* | 1.40 – 1.68 | < 0.001 |
| Female Gender | 0.58* | 0.49 – 0.68 | < 0.001 |
| Race |  |  |  |
| Black compared to White | 1.33 | 1.02 – 1.74 | 0.03 |
| Asian compared to White | 1.13 | 0.88 – 1.46 | 0.32 |
| Pacific Islander compared to White | - | | |
| American Indian compared to White | 1.06 | 0.35 – 3.26 | 0.92 |
| Other compared to White | 0.80 | 0.65 – 0.98 | 0.03 |
| Ethnicity |  |  |  |
| Hispanic | 1.47 | 1.17 – 1.84 | 0.001 |
| Insurance Type |  |  |  |
| Medicaid compared to Commercial | 1.28 | 0.66 – 2.48 | 0.46 |
| Medicare compared to Commercial | 2.69 | 2.20 – 3.29 | < 0.001 |
| Other compared to Commercial | 1.42 | 0.41 – 4.95 | 0.58 |
| Managed care compared to Commercial | 3.54 | 2.76 – 4.53 | < 0.001 |
| Primary language other than English | 0.68 | 0.52 – 0.90 | 0.01 |
| Income, zip code level (FPL) | 1.00 | 0.95 – 1.05 | 0.96 |
