# Supplementary material for: The Impact of Telemedicine on Quality of Care for Patients with Diabetes After March 2020
Source: J Gen Intern Med. 2022 Jan 28;37(5):1198–203. doi: 10.1007/s11606-021-07367-3 (PMC8796744; doi:10.1007/s11606-021-07367-3)

**Appendix 2 Table 1**: Characteristics of patients with diabetes utilizing telemedicine compared to in-person care alone, before and after the beginning of the COVID-19 pandemic

| **Characteristic** | Telemedicine utilizers | | In-person care alone | |
| --- | --- | --- | --- | --- |
|  | Pre-period  6/19/19 - 3/19/20 | Post-Period 3/19/20– 12/19/20 | Pre-period  6/19/19 - 3/19/20 | Post-Period 3/19/20 – 12/19/20 |
| Number of in-person visits |  |  |  |  |
| Primary care, mean (SD) | 4 (4) | 2 (2) | 2 (2) | 1 (1) |
| Endocrine, mean (SD) | 1 (2) | 1 (1) | 1 (1) | < 1 (1) |
| Other, mean (SD) | 2 (4) | 2 (4) | 2 (4) | 1 (3) |
| Number of telemedicine visits |  |  |  |  |
| Primary care, mean (SD) | 1 (1) | 1 (2) | 0 (0) | 0 (0) |
| Endocrine, mean (SD) | 0 (0) | 1 (1) | 0 (0) | 0 (0) |
| Other, mean (SD) | 0 (0) | 1 (2) | 0 (0) | 0 (0) |
| Telephone visits^^ |  |  |  |  |
| Primary care, mean (SD) | 1 (1) | 1 (1) | 0 (0) | 0 (0) |
| Endocrine, mean (SD) | 0 (0) | 0 (1) | 0 (0) | 0 (0) |
| Other, mean (SD) | 0 (0) | 1 (2) | 0 (0) | 0 (1) |

**Appendix 2 Figure 1:**


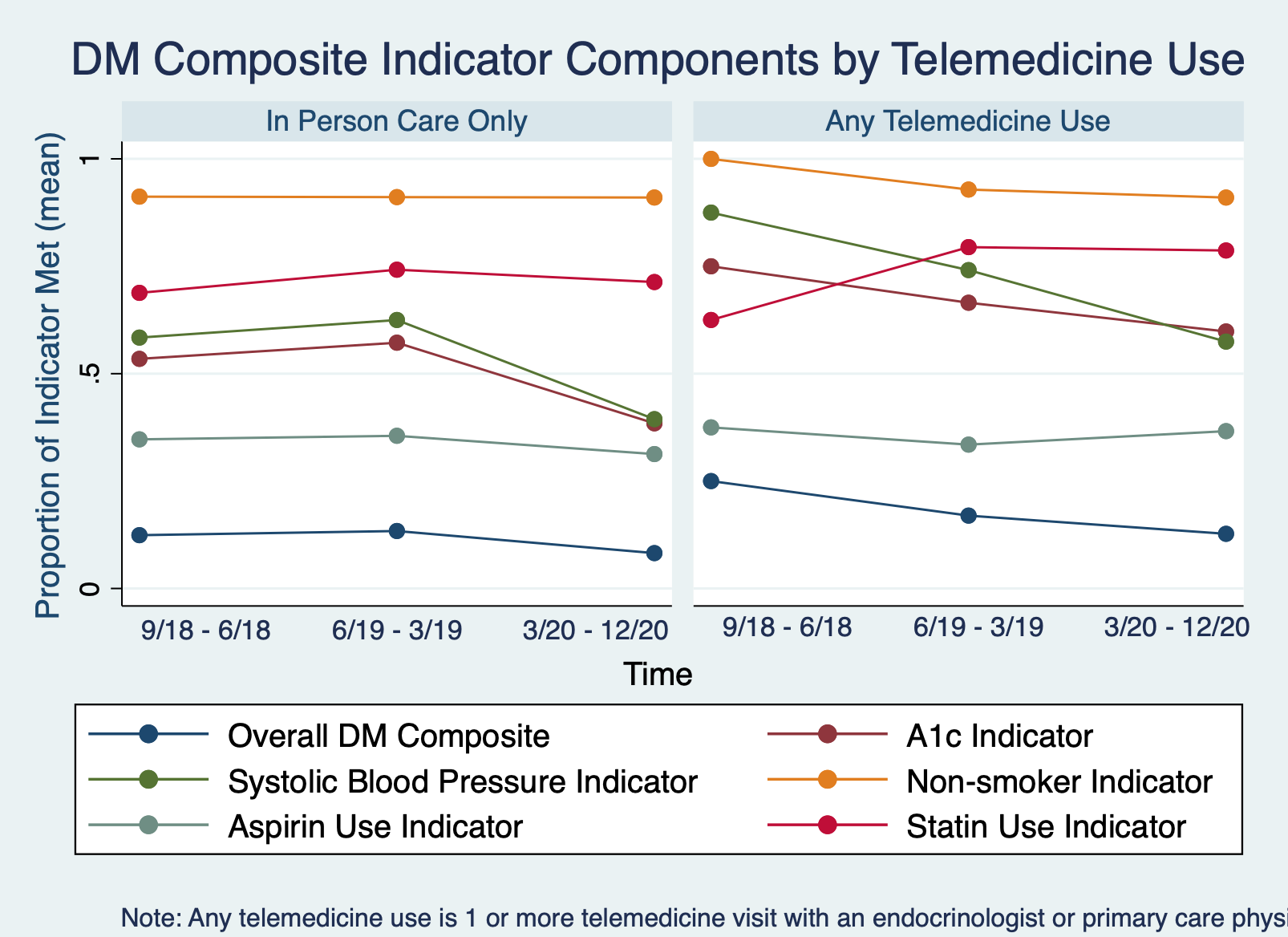

Supplement: Supplementary file 2 — (DOCX 7348 kb) [file 11606_2021_7367_MOESM2_ESM.docx]
